# Improving on and assessing ethical guidelines for digital tracking and tracing systems for pandemics

**DOI:** 10.1007/s10676-020-09561-z

**Published:** 2020-10-03

**Authors:** Björn Lundgren

**Affiliations:** 1 Department of Historical, Philosophical and Religious Studies, Umeå University, Umeå, Sweden; 2 Institute for Futures Studies, Stockholm, Sweden; 3 Department of Philosophy, Stockholm University, Stockholm, Sweden

**Keywords:** SARS-CoV-2, COVID-19, Pandemic, Guidelines, Data protection, Privacy

## Abstract

So-called digital tracking and tracing systems (DTTSs) have been proposed as a means to prevent the spread of SARS-CoV-2. There are ethical guidelines and evaluations of such systems available. As part of a research project, I will build on and critically evaluate the foundations of such guidelines. The goal is to provide both incremental improvements of the specific requirements for DTTSs and to present and discuss more fundamental challenge, the risk for indirect effects and slippery slopes. The nature of slippery slopes makes ethical guidelines more difficult since it requires a more complex analysis than, for example, using a checklist allows for.

## Introduction

After first being officially reported in December 2019, the virus SARS-CoV-2 spread within months through the world, infecting millions of people with COVID-19. At the time of writing, scientists around the world are researching and testing cures, vaccines, and improved tools for tracking, tracing, and containing the virus. All these endeavors are necessary to save lives and to re-open the global economy as well as local societies; but none of them is without ethical challenges. This research statement concerns the analyses of the ethical challenges of digital tracking and tracing systems (DTTSs), by which I broadly mean any digital application or device readily usable for viral exposure (or contact) tracing and/or notification.

Unlike vaccines and cures, many DTTSs are already fully available—while others are being developed—and the ethical evaluation and guidelines have been prompt (see, e.g., Howell O'Neill et al. 2020; Morley et al. 2020; Raskar et al. 2020). An indispensable quality of ethical guidelines for DTTSs is its usability. Specifically, guidelines should propose clear and easily understandable requirements, which can be assessed by non-experts. In this regard Morley et al. offers an excellent approach with its simple bivalent check-list criteria. Yet, there is room for improvement. In this research statement I will present a sketch of improvements of their individual requirements, but I will also present an embryo of a more serious challenge to ethical guidelines for DTTSs.

## Preliminary assessment

Morley at el.’s check-list contains two sections. The first section concerns basic permissibility of using a DTTS (what they call “go/no-go”) and contains four main questions: whether the solution is *necessary*, *proportionate*, *scientifically sound* (including whether it will be *effective*), and *temporary* (including a sunset clause).

The second section concerns an evaluation of the degree of ethical justification for the system’s properties. This section contains 12 questions. While all questions are binary (yes/no), the answers also contain descriptions for each justificatory criteria, which points to more gradual differences within the bivalent choices. The questions, or criteria, concern *voluntariness*, *consent*, *privacy and anonymity*, *self-erase function for user data*, whether the *purpose is clearly defined*, whether the *purpose is limited*, whether the system is *used only for prevention*, whether the system *monitors user’s behavior*, whether the system is *open-source*, whether the system is *equally available*, whether the system is *equally accessible*, and whether there is an *end-of-life process* which retires the system. Below I will briefly go through a few of these criteria to show the need for improvement.

Starting with the role of *effectiveness* for permissibility, how should effectiveness be evaluated in practice? A DTTS cannot be efficient for a small set of users. Should the user-base be estimated? Should the DTTS be turned-off until it reaches a sufficient user-base (risking lower willingness to use the system) or should there be a shut-off function in case it never reaches sufficient wide-spread? How should efficiency be measured? Like questions of how many must be immune to achieve heard immunity under given set of conditions, we should ask how many must use the system for it to be sufficiently efficient. Hopefully, modelling uncertainty related to these questions will decrease over time, but irrespective of the degree of factual uncertainty there is also a normative choice of what should be considered justified minimal efficiency.

The *effectiveness* requirement might also conflict with the justificatory *self-erase* *function* criterion since data deletion might affect the efficiency criteria negatively in the same way that a small user base might (e.g., if many users selectively deletes data points, especially if deletion overlaps spatiotemporally). Related to deletion, there should also be an auto-delete function, beyond the sunset clause. This is because mitigation likely would not need individual data stored longer than approximately 1 month (this varies relative to incubation period, size of the user base, etcetera).^1^

There are some issues with the consent requirement. While a consent-requirement is not wrong, it would be problematic if consent is needed. Information aggregation makes it hard, if not impossible, to foresee what can be aggregated from shared data (see, e.g., Ohm 2010; Lundgren 2020), making it difficult to properly inform an agent about the consequences of how her data will be used, violating the standard moral requirements of an informed consent (see, e.g., Eyal 2019).^2^ We also know from research on the so-called ‘privacy paradox’, that people’s reported privacy-preferences often do not correspond to their actual behavior (see, e.g., Gerber et al. 2018 for an overview), making consent somewhat of a red herring (i.e., because the presence of a consent agreement can make an ethically problematic agreement seem less problematic than it actually is). In response to this, systems should be developed so that it is likely that a reasonable agent would consent, under normal circumstances (of course, it might be difficult to determine those conditions—yet that is, partly, the aim of the other criteria).

A more serious set of problems has to do with privacy and anonymity. Morley et al. indicates a preference of locally stored data, which is sensible because then no-one has access to it unless justificatory conditions emerges. However, requiring locally stored data arguably implies, at least partly, a problematic shift in the *causal* responsibility for information security, from the operator of a DTTS solution, with centralized data storage, to the end user. Since if the sensitive data is stored locally (i.e., on the end user’s device), then the security of that data could partly depend on how the user manages her device. Thus, given that we should not hold the user *morally* responsible for securing her data when using a DTTS, local storage might be problematic.

Furthermore, Morley et al. promotes differential privacy as a means of privacy-protection. However, while differential privacy offers a technically sound way of retaining user anonymity in aggregated datasets, it does not protect against all possible harms from information aggregation. Indeed, differential privacy “addresses the paradox of learning nothing about an individual while learning useful information about a population” (Dwork and Roth 2014, p. 5), but learning useful information about a population may prove highly problematic for the individual user even if they are not individually identified—for example, since it could decrease the individual’s ability to act autonomously while retaining anonymity (Lundgren 2020). Moreover, aggregated data could reveal patterns that could be used, or misused, in ways that is harmful for individuals, groups of individuals, or society at large (ibid). In the context of DTTSs, Rasker et al. gives the example of local business, which may be associate with disease-spreading (p. 8).

Finally, another justificatory criterion promotes the idea that individuals should *only* be informed “when they have been in contact with people with confirmed infection”. This would require that massive testing capabilities be available (if not, it would conflict with the *effectiveness* requirement). Yet, in the absence of sufficient testing capabilities, it is not necessarily wrong to inform people that they have been in contact with people with a relevant set of symptoms. The question is how to balance informing people that they might have been exposed to a carrier, against the risk associated with communicating uncertain information (e.g., it may cause unnecessary worry or panic, or it may deteriorate trust in the application or the organizations behind it—which in turn may result in, e.g., less efficiency overall).

These are a few examples that I will build on to improve guidelines or checklists for DTTSs based on Morley et al.’s proposal. Next, I will turn to some more fundamental problems.

## Slippery slopes

Like the pandemic, usage of DTTSs may have far reaching effects. As Morley et al. notes, measures must be temporary. However, even with a sunset clause, there is always a risk that temporary measures become permanent (see, e.g., Donohue 2000; Rentoul 2018 for some illustrative examples), or that temporary measures—which are widely adapted—change societal norms. For example, consider how quickly social media changed many individual’s norms of information distribution. Such norm changes can be extremely detrimental.

Furthermore, as Morley et al. notes, we must analyze DTTs in a context. There is a difference between using DTTSs in a liberal democratic society and an oppressive regime. Moreover, recent changes in the political landscape reveal the risk that even relatively democratic countries can change rapidly. If this happens while DTTSs are in use, that arguably complicates matters. That is, which measures that are acceptable—and the risk that such measures be abused—can quickly change after an election.

However, this issue is arguably even more complex, since we must not only ask how decisions are affected by changes within one country or what is suitable in different countries, we must also ask what effects increased surveillance in a democratic society have on surveillance in non-democratic societies. For example, we have seen how President Trump’s behavior and rhetoric about ‘fake news’ have been used by oppressive regimes (Schwartz 2019) and there is, likewise, a risk that usage of DTTSs in liberal democracies can influence the ability to—unchallenged—use and abuse DTTSs in less liberal regimes. Of course, we should recognize that with DTTSs, the current trend seems to be the other way around: that liberal democratic are taking the lead from less liberal democracies. Yet, there is still a related risk that this lends support to less democratic regimes surveillance methods. Thus, it is crucial that we enact measures that are not only efficient, but also coheres with fundamental democratic values, and contributes positively to upholding such values globally. It is worth to note that, at the time of writing, over 70 organizations, as well a long list of dignitaries, have signed a call for protecting democratic values in the wake of Covid-19, expressing a worry of measures taken by authoritative regimes because of the crises following the global pandemic (IDEA 2020).

Thus, there are at least three slippery slopes that I want to turn my attention to in this forthcoming research:

1. The risk that temporary measures become permanent and/or permanently change information distribution norms to create a new normal, a normal which may not be preferable;
2. The risk that contextual factors (such as which political parties are in control) changes in a detrimental way after measures are in place, a change that may make previously unproblematic measures problematic;
3. The risk that measures in one country negatively influences policies in another country.

The problem with slippery slopes is that they consist of direct and indirect effects, effects which may be difficult to both identify and distinguish. It will, therefore, be challenging to draw a distinctive line between a measure that blocks one from *slipping down the slope* and one that does not. Hence, granted the risk of slippery slopes, it seems that ethical guidelines for DTTSs does not easily lend itself to check-lists, or other forms of easily determinable guidelines that can be used by a non-expert. This is part of the challenge which I hope to address in my upcoming research.

## Footnotes

1 Of course, we can imagine that study of patterns might yield information that itself can be used for mitigation purposes. But that would complicate privacy and anonymity challenges (see, e.g., Lundgren 2020).

2 There is also a risk of violation of GDPR (article 7), because of the requirement that consent requests needs to be specific (which may pose a problem, due to the informative expansion of big data analyses).
